# *spargel*, the *PGC*-*1α* homologue, in models of Parkinson disease in *Drosophila melanogaster*

**DOI:** 10.1186/s12868-015-0210-2

**Published:** 2015-10-26

**Authors:** Eric M. Merzetti, Brian E. Staveley

**Affiliations:** Department of Biology, Memorial University of Newfoundland, 232 Elizabeth Avenue, St. John’s, NL A1B 3X9 Canada

**Keywords:** *spargel*, *PGC*-*1α*, Neurodegeneration, Parkinson disease, *Drosophila melanogaster*

## Abstract

**Background:**

Parkinson disease (PD) is a progressive neurodegenerative disorder presenting with symptoms of resting tremor, bradykinesia, rigidity, postural instability and additional severe cognitive impairment over time. These symptoms arise from a decrease of available dopamine in the striatum of the brain resulting from the breakdown and death of dopaminergic (DA) neurons. A process implicated in the destruction of these neurons is mitochondrial breakdown and impairment. Upkeep and repair of mitochondria involves a number of complex and key components including *Pink1*, *Parkin*, and the PGC family of genes. *PGC*-*1α* has been characterized as a regulator of mitochondria biogenesis, insulin receptor signalling and energy metabolism, mutation of this gene has been linked to early onset forms of PD. The mammalian PGC family consists of three partially redundant genes making the study of full or partial loss of function difficult. The sole *Drosophila melanogaster* homologue of this gene family, *spargel* (*srl*), has been shown to function in similar pathways of mitochondrial upkeep and biogenesis.

**Results:**

Directed expression of *srl*-*RNAi* in the *D. melanogaster* eye causes abnormal ommatidia and bristle formation while eye specific expression of *srl*-*EY* does not produce the minor rough eye phenotype associated with high temperature *GMR*-*Gal4* expression. *Ddc*-*Gal4* mediated tissue specific expression of *srl* transgene constructs in *D. melanogaster* DA neurons causes altered lifespan and climbing ability. Expression of a *srl*-*RNAi* causes an increase in mean lifespan but a decrease in overall loco-motor ability while induced expression of *srl*-*EY* causes a severe decrease in mean lifespan and a decrease in loco-motor ability.

**Conclusions:**

The reduced lifespan and climbing ability associated with a tissue specific expression of *srl* in DA neurons provides a new model of PD in *D. melanogaster* which may be used to identify novel therapeutic approaches to human disease treatment and prevention.

## Background

Parkinson disease (PD) is a common and progressive neurological disease that is estimated to afflict 1 % of all individuals over the age of 60 years worldwide [1]. Although the direct cause of PD is as of yet unknown, the clinical symptoms include resting tremor, bradykinesia, rigidity and postural instability. In addition to these classical symptoms recent discoveries have shown that effects may include cognitive impairment such as loss of memory and depression [2]. These symptoms are caused by a decrease in the amount of dopamine available in the striatum area of the brain and in many cases characterized by an accumulation of harmful protein aggregates known as Lewy bodies in the neurons of the *substantia nigra pars compacta* [3]. The eventual dysfunction and breakdown of these neurons is responsible for the symptoms and pathology of PD [4]. Both genetic and environmental factors have been found to contribute to PD with many forms of the disease being attributed to a combination of the two. Environmental factors include: chemical exposure, brain trauma, obesity, diabetes and age [5]. Alternatively, there have been a number of familial cases of PD identified which suggests a genetic link to certain forms of this disease [6]. This link is especially strong in the case of early onset PD which shows a bias towards genetic causes, while environmental conditions seem to more often than not be linked to late onset PD [2]. Identifying and analysing the genes responsible for these inherited forms of PD may give rise to mechanisms of disease progression that are not presently understood. Impaired neuronal activity has been shown to contribute to the lack of dopamine commonly associated with PD etiology. Identifying the cause of this neuronal impairment may help lead to new, effective, strategies to combat PD.

As important and necessary components of eukaryotic cells, mitochondria are integral components in the creation of adenosine tri-phosphate (ATP) for use as chemical energy and are involved in other important pathways such as the control of cellular growth and death, cell signalling and differentiation [7]. As the mitochondria are involved in such diverse and important cellular functions; it is not surprising that the breakdown or dysfunction of mitochondria may result in a number of disorders or diseases including but not limited to movement disorders such as PD [8]. Many of these disorders are caused by defective removal of damaged or non-functional mitochondria and a subsequent lack of de novo synthesis of new mitochondria to replace the aforementioned damaged organelles. Thus, the study of genes that regulate and monitor the health of mitochondria is a reasonable next step for determining potential disease prevention strategies.

The peroxisome proliferation activated co-receptor gamma (PCG) family of genes have been linked to mitochondrial biogenesis [9]. *PGC*-*1α* has been found to be involved in de novo mitochondrial synthesis in various tissues including the liver and brain [10]. Medical data has shown that polymorphisms in *PGC*-*1α* have been commonly found in patients with early onset and severe PD [11]. Other members of this gene family, *PGC*-*1β* [12] and *PRC* (PGC-1-related-cofactor) [13] have been shown to maintain some functional homology with *PGC*-*1α* and protein sequence comparison of these genes shows close similarity and conservation of active sites [14] making it difficult to completely study the loss of function in human cells.

*PGC*-*1α* shares a functional pathway with two previously characterised PD genes; *PINK1* and *Parkin* [15]. *Parkin* is a component of a multi-protein E3 ubiquitin ligase that leads to the ubiquitination and subsequent destruction of cellular proteins [16]. Mutations to the *Parkin* gene result in the degeneration of dopaminergic (DA) neurons, most likely by allowing the aggregation of multiple dysfunctional mitochondria that eventually lead to overall cell death [8]. *PINK1* (PTEN induced putative kinase 1) is a serine/threonine-protein kinase that acts by recruiting *Parkin* to damaged mitochondria [17]. Similarly to *Parkin*, mutations in *PINK1* lead to degeneration and dysfunction of dopaminergic neurons [18]. *Parkin* and *PINK1* act together in concert along with *mitofusin 2* (*Mfn2*) to remove any damaged or dysfunctional mitochondria that may be present while *PGC*-*1α* activity regulates the creation of new mitochondria to replace damaged or removed organelles [19]. Mutation to any of the components of this pathway can lead to impaired mitochondria, decreased cellular fitness and eventual cell death.

A single PGC family gene homologue, *spargel* (*srl*), has been identified in *Drosophila melanogaster*. The SRL protein has been characterized as a downstream component of the insulin signalling TOR pathway and *srl* mutant flies have a “lean” phenotype typical of mutations that affect growth and proliferation and reduced mitochondrial fitness [20]. Although ubiquitous overexpression of *srl* has been found to negatively impact organism survival, tissue specific *srl* expression has been found to provide beneficial effects. Overexpression of *srl* has been shown to be sufficient to increase mitochondrial activity and mediate tissue specific lifespan extension in the digestive tract and intestine [21]. Altered expression of *srl* in the heart has been shown to increase capacity for exercise based endurance improvement while decreased *srl* in cardiac muscle decreases the loco-motor and endurance ability of flies [22]. The lack of gene redundancy present in *D. melanogaster* makes it an ideal model system to determine the effects of reduced or increase levels of *srl* expression on whole organism and neuronal longevity, leading to a new model of PD for use in future therapeutic studies.

## Results

**Fig. 1:**
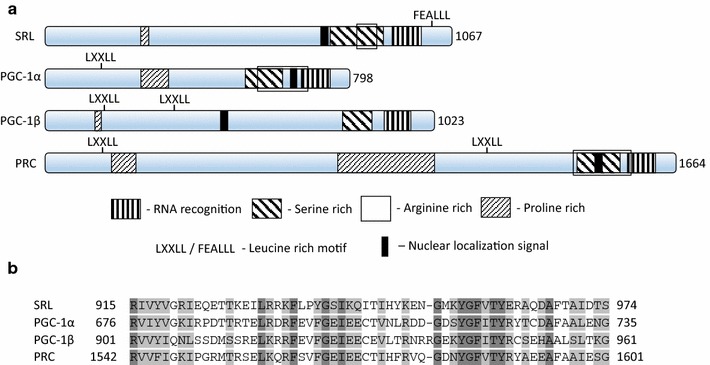
The PGC mammalian family and the *Drosophila melanogaster* protein SRL share conserved protein domains. **a** Aligned sequences show the position of each domain in one *D. melanogaster* and three human PGC family protein sequences. *Light upward diagonal* indicates proline rich region, *wide downward diagonal* indicates serine rich region, *dark vertical* indicates RNA recognition region, *solid black box* indicates nuclear localization sequence, *black frame* indicates arginine rich region, LXXLL and FEALLL indicate leucine rich nuclear recognition motifs. **b** A multiple alignment between PGC-1α, PGC-1β, PRC and SRL shows a high degree of sequence conservation within the RNA recognition motif found at the carboxyl terminus of each protein. Domains were identified using ScanProsite [41], alignment was done using ClustalW2 [42]. Protein sequences obtained from UniProt, accession numbers [Uniprot NP_037393 (PGC-1α)], [Uniprot NP_573570 (PGC-1β)], [Uniprot NP_055877 (PRC)] and [Uniprot NP_730835 (SRL)]. Elements of this figure were adapted from Scarpulla et al. [43]

A multiple alignment of the SRL protein with the three mammalian homologues; PGC-1α, PGC-1β and PRC, provides evidence of evolutionarily conserved protein structure between the human and *D. melanogaster* forms of this gene (Fig. 1). These proteins differ in length from 1664 amino acids in the case of PRC to 798 amino acids for PGC-1α but functional domains remain consistent across all four. Each contains an N terminal proline rich domain, a bipartite nuclear localization signal, C terminal serine rich region and a highly conserved RNA recognition motif as well as an arginine rich region in all except *PGC*-*1β* (Fig. 1). Each of the mammalian proteins contain at least one leucine rich motif (LXXLL) known to interact with nuclear receptors [23] that is not found in *srl*, however, an alternative leucine rich motif (FEALLL) is present which has been shown to also interact with nuclear receptors and serve the same function in *D. melanogaster* [24]. The similarities between *D. melanogaster* and mammalian proteins indicate that SRL is an ideal candidate to study PGC family activity while avoiding the functional redundancy found in other systems.

**Fig. 2:**
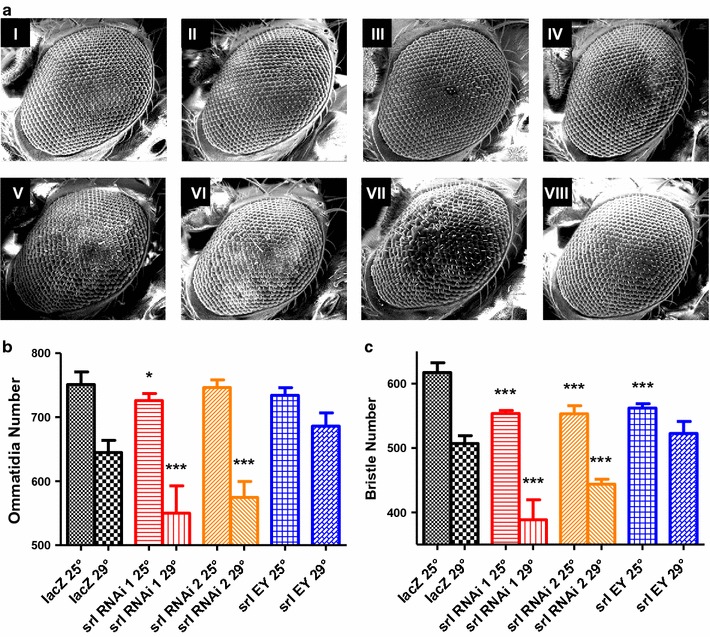
Tissue specific *srl* expression in the *Drosophila melanogaster* eye results in a reduction in both ommatidia and bristle number. **a** Scanning electron micrographs of *D. melanogaster* eyes taken at a horizontal field width of 500 µm. Genotypes are as follows: (*I*) *GMR*-*Gal4/UAS*-*lacZ* 25 °C, (*II*) *GMR*-*Gal4/srl*-*RNAi 1* (*UAS*-*srl* ^*HMS00857*^) 25 °C, (*III*) *GMR*-*Gal4/srl*-*RNAi 2* (*UAS*-*srl* ^*HMS00858*^) 25 °C, (*IV*) *GMR*–*Gal4*/*srl*-*EY* (*UAS*-*srl* ^*EY05931*^) 25 °C, (*V*) *GMR*-*Gal4/UAS*-*lacZ* 29 °C, (*VI*) *GMR*-*Gal4/srl*-*RNAi 1* (*UAS*-*srl* ^*HMS00857*^) 29 °C, (*VII*) *GMR*-*Gal4/srl*-*RNAi 2* (*UAS*-*srl* ^*HMS00858*^) 29 °C, (*VIII*) *GMR*–*Gal4*/*srl*-*EY* (*UAS*-*srl* ^*EY05931*^) 29 °C. Images were taken with a FEI MLA 650. **b** Flies show a decrease in the mean number of ommatidia present when *srl*-*RNAi* *1* (*UAS*-*srl* ^*HMS00857*^) and *srl*-*RNAi* *2* (*UAS*-*srl* ^*HMS00858*^) are driven with the *GMR*-*Gal4* driver in both standard conditions (25 °C) and at a higher temperature (29 °C). Flies show a slight but not significant decrease in ommatidia number when *srl*-*EY* is expressed in a tissue specific manner (*UAS*-*srl* ^*EY05931*^) in both standard conditions (25 °C) and at a higher temperature (29 °C). **c** Flies show a strong decrease in bristle number in standard conditions (25 °C) when *srl*-*RNAi* *1* (*UAS*-*srl* ^*HMS00857*^), *srl*-*RNAi 2* (*UAS*-*srl* ^*HMS00858*^) and *srl*-*EY* (*UAS*-*srl* ^*EY05931*^) are expressed in *D. melanogaster* eyes. At a higher temperature (29 °C) *srl*-*EY* causes an increase in number of bristles formed compared to *lacZ* controls, however, this is not statistically significant (*UAS*-*lacZ*). Comparisons were measured using a one-way ANOVA and significance was tested using a Tukey post hoc test, n = 10. *P < 0.05, **P < 0.01, ***P < 0.001

In order to assay the effect of altered *srl* activity in neurons, we induced expression of three constructs previously used by Mukherjee and Duttaroy [25]; *srl*-*RNAi* (*UAS*-*srl*^*HMS00857*^), *srl*-*RNAi* (*UAS*-*srl*^*HMS00858*^) and a *srl*-*EY* (*UAS*- *srl*^*EY05931*^) in the neuron rich *D. melanogaster* eye. Eyes develop two separate yet equally important tissues which can be assayed for neuronal loss during development; ommatidia and bristles. At 25 and 29 °C (the latter for increased expression) under the direction of *GMR*-*Gal4*, expression of both *srl*-*RNAi* transgenes decreases the number of ommatidia and bristles present in the eye (Fig. 2). Tissue specific expression of *srl*-*EY* results in a slight decrease in number of ommatidia and bristles at 25 °C and an increase in number of ommatidia and bristles at 29 °C. Similarly, *UAS*-*lacZ* under the control of *GMR*-*Gal4* shows a slight rough eye phenotype at 29 °C which is not produced by the expression of *srl*-*EY*.

**Fig. 3:**
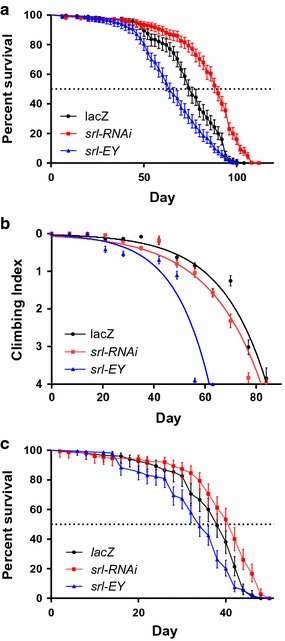
Tissue specific altered *srl* expression in dopaminergic neurons can lead to a model of Parkinson disease *Drosophila* *melanogaster*. **a** Expression of *srl*-*RNAi* (*UAS*-*srl* ^*HMS00858*^) driven by *Ddc*-*Gal4* results in an increase in lifespan compared to *UAS*-*lacZ* controls. Dopaminergic *srl*-*EY* (*UAS*-*srl* ^*EY05931*^) expression resulted in a decrease in lifespan compared to *UAS*-*lacZ* controls. Longevity is shown as a percent survival (P < 0.05 as determined by the Mantel-Cox Log Rank test) N ≥ 200. **b** Expression of *srl*-*RNAi* (*UAS*-*srl* ^*HMS00858*^) and *srl*-*EY* (*UAS*-*srl* ^*EY05931*^) driven by *Ddc*-*Gal4* cause a decrease in climbing ability over time. Dopaminergic specific expression of *srl*-*EY* causes a more severe decrease, however, *srl*-*RNAi* expressing flies live longer and climb slightly worse than *UAS*-*lacZ* controls indicating a potential decrease in climbing activity compared to lifespan. Climbing ability was determined via nonlinear curve fit (CI 95 %). *Error bars* indicate standard error of the mean, n = 50. **c** Expression of *srl*-*RNAi* (*UAS*-*srl* ^*HMS00858*^) driven by *Ddc*-*Gal4* ^HL4.3D^ at 29 °C results in an increase in lifespan while *srl*-*EY* (*UAS*- *srl* ^*EY05931*^) expression at 29 °C resulted in a decrease in lifespan compared to *UAS*-*lacZ* controls. Longevity is shown as a percent survival (P < 0.0001 as determined by the Mantel-Cox Log Rank test) N ≥ 150

To determine if the phenotype caused by altered *srl* expression found in the eye is conserved in all neurons we next looked at tissue specific expression of *srl* in the DA neurons. To assay the effect of altered *srl* activity in DA neurons, we conditionally expressed a *srl*-*RNAi* transgene (*UAS*-*srl*^*HMS00857*^) and a tissue specific *srl*-*EY* construct under the control of the *Ddc*-*Gal4* driver. Under the control of *Ddc*-*Gal4*, *srl*-*RNAi* expression leads to a marked increase in median lifespan with a premature loss of climbing ability over time when compared to the *UAS*-*lacZ* controls (Fig. 3). Tissue specific expression of *srl*-*EY* lead to both a decrease in lifespan and loco-motor climbing ability under the control of the *Ddc*-*Gal4* driver. This indicates that altered *srl* expression responds in a tissue specific nature, even within similar cell types such as neurons. We obtained somewhat similar results when we carried out the experiment at 29 °C, indicating that the observed differences in phenotype between eye and dopaminergic neurons was tissue and not temperature specific.

## Discussion

*PGC*-*1α* has been identified as a modulator of mitochondrial biogenesis, energy metabolism [26], insulin signalling [27] and is believed to be linked to human ailments including Alzheimer [28], Huntington [29] and Parkinson diseases [30]. The study of human *PGC*-*1α* is complicated by the partial functional redundancy of the other PGC family members, *PGC*-*1β* and *PRC* (PGC-1-related-cofactor). The single Drosophila PGC family homologue, *srl*, has been linked to mitochondrial biogenesis and insulin signalling [25]. We investigate *srl* as a potential key regulator of disease progression and seek to model this system for future analysis.

The *D. melanogaster* eye has been used to study potential genes involved in neurodegenerative disease due to the neuron rich nature of the developing tissues located there. When two *srl*-*RNAi* constructs are expressed in this tissue, there is a significant reduction in the number of ommatidia and bristles formed. This effect is exacerbated when flies are raised at 29 °C, caused by an increase in the activity of the Gal4 expression system. Alternatively, expression of a previously characterized *srl*-*EY* transgene seems to have little effect on ommatidial viability under normal physiological conditions (25 °C). At 29 °C the amount of ommatidial degeneration increases significantly across all genotypes. Interestingly, overexpression of *srl*-*EY* at 29 °C does not cause significant degeneration of ommatidia or bristles as found in the *UAS*-*lacZ* control at higher temperatures. Experiments by Mukherjee and colleagues have shown that *srl* overexpression can rescue FoxO mediated eye destruction, indicating a similar effect [25]. It is possible that tissue specific expression of *srl* in the eye may be sufficient to prevent physiological stressors from causing aberrant cell formation under these conditions.

Surprisingly, expression of *srl*-*RNAi* in DA neurons under the control of the *Ddc*-*Gal4* driver caused an increase in the mean lifespan of flies. Despite the increased longevity of these flies, they lose their climbing ability slightly earlier than controls. When considering the increase in lifespan this indicates that although they live longer, they may have severe loco-motor defects during the latter part of their life. Alternatively, DA expression of the *srl*-*EY* transgene under the control of the *Ddc*-*Gal4* driver showed a significant decrease in lifespan compared to *UAS*-*lacZ* controls. The loco-motor and climbing ability of these flies was also decreased. Taken together, the premature mortality and decreased climbing ability displayed in flies expressing *srl*-*EY* in dopamine decarboxylase neurons appears to give a new, previously uncharacterized, model of PD in *D. melanogaster*.

Altered *srl* expression has been found to cause various phenotypes in a tissue specific manner. Ubiquitous overexpression of *srl* has been shown to moderately reduce mean lifespan while overexpression in intestinal stem cells and cells of the digestive tract caused increased lifespan [21]. Similarly, increased *srl* expression in cardiac muscle has been linked to exercise based endurance improvement and cardiovascular performance [22]. These findings lead us to believe that altered *srl* expression does not react the same way in the eye model of neurodegeneration as in DA neurons.

Although an increase in lifespan caused by the expression of *srl*-*RNAi* was an unexpected result, we hypothesize that a strong decrease in *srl* expression causes an amount of mitochondrial stress sufficient to activate a protein stress response which has been shown to increase lifespan [31]. The exact mechanism of this increase is a topic of much debate with the most popular hypothesis involving an activation of the unfolded protein response (UPR) [32]. However, it has recently been found that in *Caenorhabditis elegans* many of the genes involved in the UPR are non-essential for this increase in longevity, suggesting another mechanism for the increase in organismal longevity [33]. Alternatively, this increase in longevity may involve the concept of stress causing the formation of reactive oxygen species (ROS) at a low level which provoke cellular anti-oxidants, causing a stronger response to future ROS exposure [34]. The most commonly used term for this is mitochondrial hormesis (or mitohormesis) [35]. It is quite plausible that stress causes increased longevity through a combination of factors involving ideas from both of the aforementioned explanations.

## Conclusion

The use of a model organism in identifying and characterizing the pathways of disease progression is a fundamental step in creating new and novel treatment options. Studying the consequences of altered *srl* expression in *D. melanogaster* allows us to study the complex mammalian PGC gene family in a system containing only a single homologue. Identifying the role of *srl* will lead to an understanding of the associations and pathways related to proper function of this gene and subsequently the consequences of improper gene function. Currently, there is no preventative treatment available for PD with limited options available to combat advanced symptoms. Identification of this new model of PD provides a framework for more advanced studies into complex gene interactions. Connecting cellular processes and characterizing genetic pathways of disease progression may eventually allow for the preventative treatment of genetic forms of PD and novel therapeutic options.

## Methods

### Drosophila media

The standard cornmeal-yeast-molasses-agar medium is made with 65 g/L cornmeal, 10 g/L nutritional yeast and 5.5 g/L agar supplemented with 50 mL/L fancy grade molasses and 5 mL of 0.1 g/mL methyl 4-hydroxybenzoate in 95 % ethanol and 2.5 mL of propionic acid in standard plastic shell vials. The medium is stored at 4–6 °C and warmed to room temperature for use.

### Drosophila transgenic lines

To express transgenes in a subset of cells, including the dopaminergic neurons, the *Ddc*-*Gal4*^*HL4.3D*^ line was the generous gift of Dr. Jay Hirsh (University of Virginia) [36]. The following lines were obtained from the Bloomington Drosophila Stock Center at Indiana University-Bloomington: (1) to drive expression behind the morphogenetic furrow in the developing eye disc *GlassMultipleReporter*-*Gal4*^12^ (*GMR*-*Gal4*; [37]); (2) to act as a benign control for the ectopic expression of transgenes *UAS*-*lacZ*^*4*−*2*−*1*^ (*UAS*-*lacZ*; [38]); (3) to express in the presence of Gal4 the endogenous *srl* gene product a line bearing an *EPgy2* insertion in the 5 prime flanking region of *srl: y w; P{EPgy2*^*srlEY05931*^ (*UAS*-*srl*^*EY05931*^) and (4) to, in the presence of Gal4, express a dsRNA for RNA inhibition (RNAi) of *srl*: *y sc v; P{TRiP.HMS00857}attP2* (*UAS*-*srl*^*HMS00857*^) and *y sc v; P{TRiP.HMS00858}attP2* (*UAS*-*srl*^*HMS00858*^).

### Scanning electron microscopy of *Drosophila melanogaster* eye

Female virgins of the *GMR*-*Gal4* were mated with *UAS*-*lacZ*, *UAS*-*srl*^*EY05931*^, *UAS*-*srl*^*HMS00857*^ and *UAS*-*srl*^*HMS00858*^ males. Male progeny of each cross were collected, aged for 3–5 days and frozen at −80 °C. Flies were mounted under a dissecting microscope, and desiccated overnight. The eyes of mounted flies were imaged via scanning electron micrography at 130 times magnification with a Mineral Liberation Analyzer 650F scanning electron microscope. Total bristle count, and total ommatidia count were obtained using ImageJ [39].

### Ageing analysis

Female virgins of the *Ddc*-*Gal4* line were mated with *UAS*-*lacZ*, *UAS*-*srl*^EY05931^ and *UAS*-*srl*^HMS00858^ males. Male progeny of each cross were collected, maintained in cohorts of no more than 20 to avoid crowding and were placed on new medium every 2 or 4 days for the duration of the experiment. Flies were scored for viability every 2 days until all flies in all genotypes perished. Survival curves were compared by the log-rank (Mantel Cox) test.

### Loco-motor analysis

From each of the crosses described above, fifty male progeny were collected and maintained in vials of ten flies, and transferred to new medium twice weekly throughout the duration of the experiment. One week (7 days) after collection, and in seven-day intervals, five cohorts of flies for each genotype were assessed for climbing ability as previously described [40]. Flies were scored every 7 days for their ability to climb within a glass tube of 1.5 cm diameter. Ten trials of each cohort of ten or less flies were scored based upon two cm intervals of height reached. A climbing index was calculated for each vial by the equation: climbing Index = ∑ nm/N, where n is the number of flies at a given level, m is the score for that level (1–5), and N is the total number of flies climbed for that trial. A nonlinear regression curve of 95 % confidence intervals was used to analyze graphs of 5—climbing index as a function of time in days for each genotype. The slope (k) and Y-intercept (Y°) of each non-linear regression curve were calculated, where slope represents the rate of decline in climbing ability, and the Y-intercept represents the initial climbing ability (in the form of 5—climbing index). As neither the slope nor the Y-intercept remained constant across all groups, it was necessary that both parameters were incorporated into statistical analysis to determine differences in climbing ability. A comparison of fits concluded whether or not curves differed between groups.

## Abbreviations

ATP: adenosine tri-phosphate
DA: dopaminergic
*D. melanogaster*: *Drosophila melanogaster*
Mfn2: mitofusin 2
PD: Parkinson disease
PRC: PGC-1 related cofactor
PGC: proliferation activated co-receptor gamma
Pink1: PTEN induced putative kinase 1
srl: spargel
